# Modification of a Carboxymethyl Cellulose/Poly(vinyl alcohol) Hydrogel Film with Citric Acid and Glutaraldehyde Crosslink Agents to Enhance the Anti-Inflammatory Effectiveness of Triamcinolone Acetonide in Wound Healing

**DOI:** 10.3390/polym16131798

**Published:** 2024-06-25

**Authors:** Kanticha Pratinthong, Winita Punyodom, Pensak Jantrawut, Kittisak Jantanasakulwong, Wirongrong Tongdeesoontorn, Montira Sriyai, Rangsan Panyathip, Sarinthip Thanakkasaranee, Patnarin Worajittiphon, Nuttapol Tanadchangsaeng, Pornchai Rachtanapun

**Affiliations:** 1 Division of Packaging Technology, School of Agro-Industry, Faculty of Agro-Industry, Chiang Mai University, Chiang Mai 50100, Thailand; kanticha_p@cmu.ac.th (K.P.); kittisak.jan@cmu.ac.th (K.J.); rangsanpanyatip@gmail.com (R.P.); sarinthip.t@cmu.ac.th (S.T.); 2 Department of Chemistry, Faculty of Science, Chiang Mai University, Chiang Mai 50200, Thailand; patnarin.w@cmu.ac.th; 3 Center of Excellence in Materials Science and Technology, Faculty of Science, Chiang Mai University, Chiang Mai 50200, Thailand; 4 Department of Pharmaceutical Sciences, Faculty of Pharmacy, Chiang Mai University, Muang, Chiang Mai 50200, Thailand; pensak.j@cmu.ac.th; 5 Center of Excellence in Agro Bio-Circular-Green Industry (Agro BCG), Chiang Mai University, Chiang Mai 50100, Thailand; 6 School of Agro-Industry, Mae Fah Luang University, 333 Moo 1 Tasud, Chiang Rai 57100, Thailand; wirongrong.ton@mfu.ac.th; 7 Research Center of Innovative Food Packaging and Biomaterials Unit, Mae Fah Luang University, 333 Moo 1 Tasud, Chiang Rai 57100, Thailand; 8 Office of Research Administration, Chiang Mai University, Chiang Mai 50200, Thailand; montirasriyai@gmail.com; 9 Bioplastics Production Laboratory for Medical Applications, Faculty of Science, Chiang Mai University, Chiang Mai 50200, Thailand; 10 College of Biomedical Engineering, Rangsit University, Pathumthani 12000, Thailand; nuttapol.t@rsu.ac.th

**Keywords:** hydrogel, carboxymethyl cellulose, poly (vinyl alcohol), crosslinking, triamcinolone acetonide

## Abstract

Anti-inflammatory wound healing involves targeted drug delivery to the wound site using hydrogel materials to prolong drug effectiveness. In this work, hydrogel films were fabricated using carboxymethyl cellulose (CMC) and poly(vinyl alcohol) (PVA) crosslinked with citric acid (CA) and glutaraldehyde (GA) at different concentrations. The crosslinker densities were optimized with various CA (2–10% *w*/*v*) and GA (1–5% *v*/*v*) concentrations. The optimized crosslink densities in the hydrogel exhibited additional functional group peaks in the FT-IR spectra at 1740 cm^−1^ for the C=O stretching of the ester linkage in CA and at 1060 cm^−1^ for the C-O-C stretching of the ether group in GA. Significantly, the internal porous structures of hydrogel composite films improved density, swelling capacities, solubility percentage reduction, and decreased water retention capacities with optimized crosslinker densities. Therefore, these hydrogel composite films were utilized as drug carriers for controlled drug release within 24 h during medical treatment. Moreover, the hydrogel films demonstrated increased triamcinolone acetonide (TAA) absorption with higher crosslinker density, resulting in delayed drug release and improved TAA efficiency in anti-inflammatory activity. As a result, the modified hydrogel showed the capability of being an alternative material with enhanced anti-inflammatory efficiency with hydrogel films.

## 1. Introduction

Hydrogels are hydrophobic polymers with a lattice molecular structure, revealing an ability to retain a large amount of water or liquid within the structure, showing softness and flexible features similar to tissues in the body. Therefore, hydrogels are used as a material in various medical treatment applications such as drug delivery and wound dressing. Interestingly, the promising properties of hydrogels were reported by using them to deliver antibiotics to any body site and control the drug release at the desired site, showing better efficiency in biocompatibility than traditional drug delivery systems [1]. Carboxymethyl cellulose (CMC) is a cellulose derivative produced by the reaction of sodium monochloroacetate with alkali cellulose [2]. CMC is dissolved in water and can be used as a stabilizer to increase the viscosity of binding agents in food [3], for example, when mixed into ice cream and pudding. However, the viscosity and stability of CMC are dependent on pH levels being between 5–10 and there is a loss of stable properties at pH levels lower than 5 [4]. In general, CMC is widely used in various industries, such as the washing, paint, adhesive, textile, paper, ceramic, food, and pharmaceutical industries, because of its properties, such as being a white solid, odorless, tasteless, and environmentally friendly as it is water-soluble [5]. The high viscosity of CMC can assist with adhesion and as a stabilizer, meaning that it can be used in the food industry as a thickening agent in ice cream, in medical materials as a coating on drug capsules, or as a gelling agent in pharmaceuticals [6].

In general, the mechanical properties of CMC films can be enhanced by blending with other polymers such as poly(vinyl alcohol) (PVA) and adding a crosslinking agent. Glutaraldehyde (GA) [7] is classified as a crosslinking agent, favoring usage in crosslinked CMC/PVA hydrogel films. The crosslinking effect on the properties of CMC/PVA films has been investigated, revealing that the addition of GA resulted in the improvement of the mechanical strength of the hydrogel film by utilizing methylene blue sorbents [8,9]. GA also induced essential features in the development of water-soluble CMC/PVA hydrogel films for use as a drug delivery material (drug release) through the crosslinking effect [9]. Furthermore, citric acid (CA) is another crosslinking agent used to improve the properties of CMC/PVA due to its low cost and non-toxic properties [10].

To study a model of drug release on CMC/PVC [11], gentamicin sulfate (GTM) was used to analyze and show the formation of the ester bond and enhanced tensile strength. Thus, the CMC/PVA hydrogel films revealed a tendency to prolong the release time of GTM above 24 h [12]. CMC/PVA hydrogels can be promised as water-soluble biomaterials for drug delivery. For the basics, PVA is a biodegradable, semi-crystalline synthetic polymer widely used in biotechnology for applications such as tissue regeneration, wound dressings, and drug delivery systems. Meanwhile, PVA hydrogels are used and integrated with many biomedical and pharmaceutical applications. They are mainly used as a drug delivery material for contact lenses, which manipulates the properties of the hydrogel material through the preparation process related to the type and concentration of crosslinks [13]. Recently, PVA and hyaluronic acid (HA) hydrogels have gained recognition as promising biomaterial systems due to their distinct properties and extensive applications in the biomedical field [14]. This research investigated the potential of the wound gel with antimicrobial effects based on PVA, integrated with a compound of silver-containing gels for wound healing through its non-toxicity, high absorption capacity, and effective wound-healing capabilities [15]. Then, the PVA/CMC copolymers were prepared by electron beam irradiation as a crosslinking agent for dye removal, which has been performed by Taleb et al. [16], and the hydrophilic copolymer was obtained from PVA/CMC hydrogels for the retention of anionic dye pollutants [16]. Thus, using PVA aims to provide non-toxic, biodegradable dressings with antimicrobial properties for effective wound care therapy [17].

Smart hydrogels represent a class of stimuli-responsive materials with promising applications in various fields, including biomedicine, environmental science, and soft robotics. This review focuses on the synthesis, properties, and potential material applications of a smart hydrogel composed of CMC grafted onto PVA [18]. The synthesis process and potential material applications of CMC-grafted PVA hydrogels, such as drug delivery systems, tissue engineering scaffolds, actuators, and sensors, offer insights into future research directions and innovations in this field. The incorporation of ester linkages between PVA and CMC chains significantly influences the properties of resulting hydrogels, including structural, mechanical, and biological characteristics. Ester-linked PVA/CMC [19] hydrogels exhibit altered swelling behavior, improved mechanical properties, and maintained biocompatibility compared to conventional hydrogels. These findings underscore the potential of ester-linked PVA/CMC hydrogels for various biomedical and material applications, warranting further exploration and optimization in future research endeavors.

Thus, it was hypothesized that the type of crosslink agent in the CMC/PVA hydrogel film can control the loading and release capacity of the soluble prototype drug. The releasing capacity of hydrogels containing triamcinolone acetonide (TAA) depended on the chemical structure and physical and thermal properties of CMC/PVA hydrogels.

TAA was defined as an anti-inflammatory drug to be applied with the hydrogel prototype medication employed in this study. For the basics, TAA is utilized in ophthalmology for managing inflammatory eye conditions such as uveitis and in rheumatology for intra-articular injections to alleviate inflammation and pain in arthritis. In addition, TAA plays a role in oral and dental medicine, targeting conditions such as oral ulcers and gingivitis. The rationale behind employing TAA for inflammation stems from its ability to modulate immune responses and attenuate inflammatory processes, which are fundamental in numerous diseases. TAA effectively alleviates symptoms and facilitates recovery in a spectrum of inflammatory disorders by mitigating inflammation.

For the TAA feature, it was chosen to test this work due to its promising properties. It shows the benefit of anti-fibrotic properties in gelatin sheets for tissue engineering using a mouse skin wound model, significantly improving wound closure with the TAA injection. In addition, the worth of TAA-loaded gelatin sheets for localized drug delivery in tissue engineering underscores their potential to enhance wound healing outcomes by effectively modulating fibrotic responses [11]. Thus, TAA was incorporated into biodegradable gelatin, processed into freeze-dried sheets, and used as the hydrogel to study TAA release kinetics. Meanwhile, other related work did not report developing an alternative hydrogel with good drug-loading performance and TAA drug-release capability as an anti-inflammatory wound dressing [20].

This work aims to study the crosslinking effects of GA and CA additive agents in various concentrations on the properties of CMC/PVA hydrogels containing TAA. It indicated the preparation approach of hydrogel films with CMC/PVA, containing prototype drugs with different concentrations of GA and CA crosslinking agents, to improve the hydrogel patch’s ability for wound healing and use as an optional medical material.

## 2. Materials and Methods

### 2.1. Materials

CMC was purchased from Union Science Co., Ltd. (Chiang Mai, Thailand). PVA (Molecular Weight: ~100,000) was supplied by Chem Supply Co., Ltd. (Gillman, Australia), CA (99.5% of purity) and the GA solution (25% concentration) were supplied by Loba Chemie Co., Ltd. (Tarapur, India), and TAA was purchased from Tianjin Tianyao Pharmaceutical Co., Ltd. (Tianjin, China).

### 2.2. Preparation of Hydrogel films

PVA was dissolved in distilled water and heated in a water bath at 50 °C for 20 min under reflux. The PVA solution was then cooled to room temperature and mixed with CMC [20,21] CA (2–10% *w*/*v*) and GA (1–5% *v*/*v*) crosslinking agents, which were added in varying concentrations. Subsequently, TAA (0.1%) was loaded into the CMC/PVA hydrogel films under each specified condition (Tables S1 and S2). The mixture was stirred with a magnetic stirrer until homogenous and left overnight in a closed compartment to remove air bubbles. The clear solution was poured into a Petri dish (9 cm^2^) and dried in a hot air oven at 50 °C for 24 h. The dried CMC/PVA/CA/TAA and CMC/PVA/GA/TAA films were cured at 80 °C for 5 min to enhance crosslinking between polymer chains. The cured films were washed with distilled water until neutral (pH: 7), resulting in swollen films. They were then washed with isopropyl alcohol to remove any remaining water within the matrix and subsequently characterized for film properties.

### 2.3. Chemical Structure

The chemical functional groups of CMC, PVA, CA, GA, and hydrogel films were examined using a Fourier-transform infrared spectroscopy (FT-IR) spectrophotometer (FT-IR, FT/IR-4700, JASCO International Co., Ltd., Pfungstadt, Germany). The samples were examined by FT-IR in the attenuated total reflectance mode (ATR), collecting signals in the 400–4000 cm^−1^ range [22,23].

### 2.4. Morphology

The morphology of the hydrogel films was analyzed using a scanning electron microscope (SEM) at 10 kV (Scanning electron microscope, model JSM-IT300LV JEOL Co., Ltd., Tokyo, Japan). The mixture solution of CMC/PVA (20 mL) was added with CA and GA at 2–10% and 1–5% concentrations, respectively, and poured into a Petri dish for a freeze-drying process to obtain the hydrogel samples. The hydrogel samples were frozen using a liquid nitrogen solution, and broken hydrogel samples were used to observe cross-sections for the lateral crosslinking of the hydrogels. Afterwards, the dried and broken hydrogel samples were mounted on stubs and coated with gold particles via a sputtering process to improve the image contrast quality. Then, the SEM images were captured [24,25].

### 2.5. Properties of Hydrogel Films

#### 2.5.1. Crosslinking

The hydrogel films (1 × 1 cm^2^) were immersed in water and stirred for 12 h to dissolve the unreacted hydrogel. The remaining hydrogels were filtered and washed in water and acetone. Then, hydrogels were dried at 40 °C overnight, and the percentage of crosslinking (crosslinking (%)) was estimated according to the following Equation (1) [23]

(1) $$Crosslinking\;(\%)=\frac{W_{1}}{W_{2}}\times 100$$

where *W*_1_ and *W*_2_ are the weights of the dried films before and after dissolution, respectively. Crosslinking (%) was averaged from 5 measurements.

#### 2.5.2. Degree of Swelling

The swelling behavior of the hydrogel films was studied in phosphate buffer (PSB) (pH 7.4) at 37 °C [21]. The hydrogel films (0.2 g) were immersed in the beaker containing 20 mL of buffer. The buffer removed the swollen film samples from the hydrogel surface to remove the excess water under 2 h, following the weight on the analytical balance. Thus, the swelling ratio of the hydrogel films was determined by using Equation (2) [25,26]

(2) $$Swelling\;Ratio=\frac{W_{s}-W_{d}}{W_{d}}$$

where *W_d_* and *W_s_* represent the weights of the dry and wet hydrogel, respectively.

#### 2.5.3. Solubility

The sample film with size 10 mm × 10 mm × 0.05 mm (width × length × thickness) was dried at 60 °C for 24 h and placed in a 250 mL Erlenmeyer flask containing 50 mL of distilled water, then shaken at 25 rpm for 24 h using a shaker. The supernatant was filtered, and the remaining samples were collected. The residue sample on the filter paper was dried in a hot-air oven at 80 °C for 24 h. Therefore, the water solubility percentage was calculated in quintuplicate using Equation (3) [27]

(3) $$Solubility(\%)=\frac{W_{1}-W_{2}}{W_{1}}\times 100$$

where *W*_1_ and *W*_2_ represent the weights of dry and wet hydrogels, respectively.

#### 2.5.4. Water Retention Capacity

The water retention property of bacterial cellulose composite sponges was obtained by immersing dry hydrogel in PBS. After 24 h, wet hydrogels were collected from the PBS and wiped with filter paper to remove excess surface PBS. Thus, the initial wet hydrogel weight (*W*_0_) was recorded and transferred to Petri dishes at room temperature. Meanwhile, wet hydrogels (*W_t_*) were measured at regular time intervals for 24 h, and water retention capacity (%) was calculated using the following Equation (4), with the test repeated 3 times [6]

(4) $$Water\;retention\;capacity\;\left(\%\right)=\frac{W_{t}}{W_{0}}\times 100$$

where *W_t_* and *W*_0_ represent the weights of the wet and dry hydrogel, respectively.

### 2.6. Release Ability of Anti-Inflammatory Drugs (In Vitro)

Square-shaped films (1 × 1 cm^2^) of CMC/PVA, CMC/PVA/CA, and CMC/PVA/GA were immersed in 20 mL phosphate buffer saline (pH 7.4) at room temperature under semi-static conditions. The dissolution medium (1 mL) was collected at each predetermined time (0.05, 0.08, 0.25, 0.5, 1, 3, 6, 12, and 24 h). TAA was used as a model drug to determine the release behavior of the hydrogel films. The dried sample was soaked in the solution to investigate drug loading. To determine the release of TAA, the preloaded hydrogel was placed in a phosphate-buffered saline solution. At time intervals, a 2 mL liquid sample was taken to be analyzed, and an equal amount of fresh buffer solution was added to maintain the sink condition. The experiment was repeated in triplicate. The amount of TAA released was detected using UV–visible spectroscopy (UV–Vis) with an absorption of 295 nm. The cumulative release of the drug was calculated based on the following Equations (5)–(7) [13]

(5) $$Concentration=\frac{Absorbant\;at\;295\;nm}{Standard\;curve}$$

(6) $$Drug\;content=\frac{Concentration}{Dilution\;factor}$$

(7) $$\%\;Release=\frac{Drug\;content\times 100}{Theory\;content}$$

### 2.7. Statistical Analysis

One-way ANOVA using SPSS (Statistical Package for the Social Sciences) software 17.0 was used to analyze the results. Statistically significant differences at a confidence interval of 95% (*p* < 0.05) were estimated using the Duncan multiple range test over five samples.

### 2.8. Kinetic Release

The kinetics of TAA release were conducted for this study with the hydrogel in different kinetic models at pH 7.4. In addition, the kinetics of release are used to consider the correlated drug diffusion models, which are defined with various kinetic models, such as the zero-order, first-order, Higuchi, and Korsmeyer–Peppas models, following Equations (8)–(11).

Zero-order:

(8) $$F=k_{o}t$$

First-order:

(9) $$ln\;(1-F)=-k_{1}t$$

Higuchi:

(10) $$F=k_{h}t^{1/2}$$

Korsmeyer–Peppas:

(11) $$M_{t}/M_{\infty }=k_{kp}t^{n}$$

where *F* is the fraction of TAA released at time *t*, *k*_0_, *k*_1_, *k_h_*, and *k_kp_* are the constants of the zero-order, first-order, Higuchi, and Korsmeyer–Peppas kinetic release models, respectively [28,29,30].

## 3. Results and Discussion

### 3.1. Characterization of Hydrogel Films

#### 3.1.1. Hydrogel Functional Structure Analysis

**Scheme 1:**
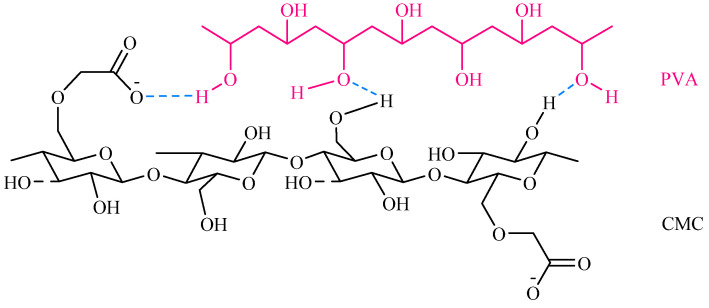
Molecular interactions of PVA and CMC [31].

**Figure 1:**
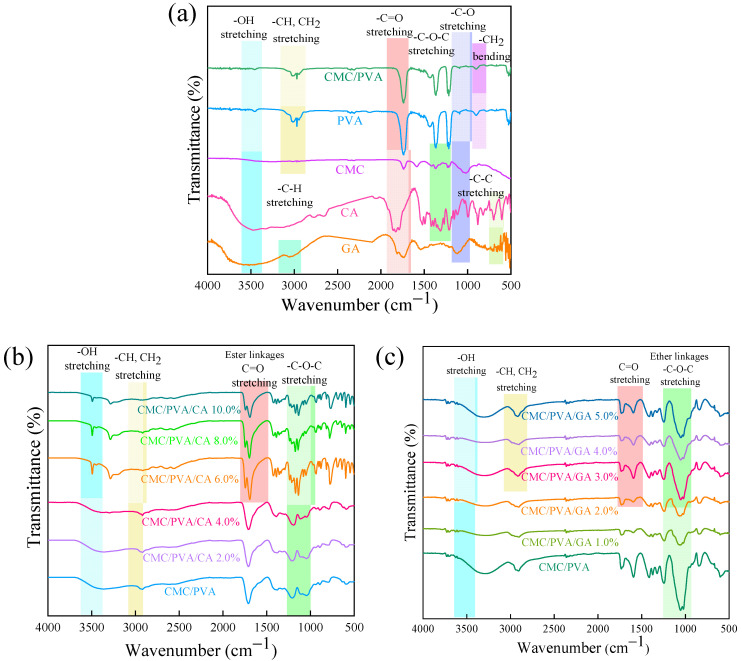
(**a**) FTIR spectra of GA, CA, CMC, PVA, CMC/PVA, and the crosslink functional groups of CMC/PVA hydrogel films with (**b**) CA and (**c**) GA in various concentrations.

As shown in Figure 1a, PVA was modified by CMC based on the hydrogen bonding between the hydroxyl groups (-OH) in PVA and the carboxymethyl groups (-CH_2_-COOH) in CMC [9]. The chemical interaction structure between PVA and CMC is illustrated in Scheme 1. CMC contains carboxyl groups (-COOH) and -OH, which can form hydrogen bonds. PVA is also mainly composed of -OH, which is also engaged in hydrogen bonding. Thus, the primary mechanism binding these two polymers in the hydrogel network is the formation of hydrogen bonds between the -OH of PVA and the -COOH or -OH of CMC. For the interaction, it was indicated that PVA exhibited a head-to-tail configuration, in which the head is more hydrophilic than the tail end. This increased hydrophilicity was enhanced by the binding capability of -OH at the head end to the CMC structure, facilitating stronger hydrogen bond formation in the PVA/CMC structure.

The effect of the crosslinking of CA and GA on the chemical functional groups of CMC/PVA hydrogel films (Scheme 1) was analyzed using FT-IR spectroscopy over the wavenumber range of 500–4000 cm^−1^. Figure 1a illustrates the predominant functional group components of CA, GA, CMC, PVA, and CMC/PVA from the FT-IR spectra, highlighting carboxyl, hydroxyl, and hydrocarbon chemical functional groups, as summarized in Table 1. Additionally, Figure 1b,c depicts the chemical functional groups of CMC/PVA hydrogels, demonstrating the enhanced crosslinking structure with the addition of CA and GA additive agents, respectively. The overall functional groups of CMC/PVA hydrogel films and their modified conditions are presented in Tables S1 and S2.

The results of carboxyl content determination were in accordance with this finding. Additionally, a noticeable shift of the carbonyl peak towards lower wavenumbers was observed with increasing amounts of PVA.

Due to the formation of hydrogen bonds, FT-IR spectra revealed a characteristic of the carbonyl stretching vibration (ν(C=O)) due to hydrogen bonding at 1650–1750 cm^−1^ [32], resulting in both broadening and a slight decrease in frequency compared to the peak observed in the absence of hydrogen bonding at 3400–3300 cm^−1^ [33]. It was indicated that the shifting of ν(C=O) suggests an enhanced hydrogen bonding interaction between the OH groups of PVA and the carbonyl groups (C=O) of the free acid and ester groups in the CMC/PVA hydrogel [21,34,35]. Consequently, the ν(C=O) peak in the FT-IR spectra indicates the presence of hydrogen bonding in the CMC/PVA hydrogel, showing the strong bonding vibration signals and strength of hydrogen bonds within the molecule. Moreover, an increase in the peak intensity at 2942 cm^−1^ was observed, indicating the presence of CH_2_ groups also associated with PVA.

**Scheme 2:**
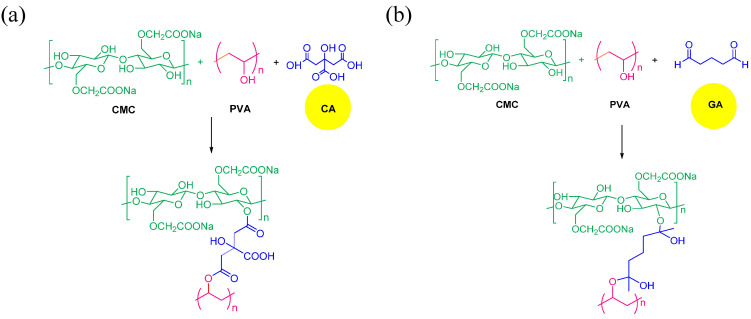
Molecular interactions of ester and ether with CMC/PVA resulting from crosslink formation via (**a**) CA and (**b**) GA, respectively.

Additionally, the CMC/PVA/CA hydrogel films displayed a new characteristic peak at 1740 cm^−1^, corresponding to the C=O stretching of the ester linkage (Scheme 2a). Figure 1c illustrates the crosslinked CMC/PVA hydrogel with GA, prepared using the solution-casting technique. CMC serves as a polyanionic polymer with a major carboxyl group (–COO^−^) [28], while PVA functions as a supporting polymer to enhance the mechanical properties and stability of CMC. Meanwhile, GA acts as a crosslinking agent, further improving the properties of the CMC/PVA hydrogel. In the CMC/PVA/GA hydrogel films, a band at 1060 cm^−1^ was observed, indicating the crosslinking reaction between PVA and GA associated with the stretching of ether groups (C–O–C). Scheme 2b demonstrates the successful preparation of the crosslinked CMC/PVA hydrogel with GA using the solution-casting technique. Based on the comparison of varying CA and GA concentrations for crosslinking agents, Scheme 2b presents the ligation of PVA and CMC polymers, which explains the increase in CH_3_ molecules in the GA-added condition. This study reveals that GA exhibits extra CH_3_ structures compared to CA with a higher concentration of CA, corresponding to the FT-IR results, as summarized in Table 2.

**Table 1:** FTIR peak assignments and their corresponding functional groups observed in CMC, PVA, CA, and GA.

Wavenumber (cm^−1^)				Characteristic Bond and Movement	Ref.
CMC	PVA	CA	GA		
3434	3480	3500 −3200	2925 −2854	O−H stretching vibration (Chain end)	[36]
-	2917	-	-	Originated from stretching vibrations of -C-O-C and –CH of aldehyde group	[31,37]
1740	-	1693	1624	C=O stretching vibration	[38]
1624	-	-	-	C–H stretching vibration	[5]
-	1425	-	1400	C–H bending in CH_2_, CH_3_	[39]
-	1325	-	-	C–H bending in CH_2_, CH_3_	[16]
-	1081	1082	1083	C–O stretching vibration	[16]
-	-	945	-	Deformation vibrations of hydroxyl group are located	[36]
-	839	-	-	C–H bending in CH, CH_2_, CH_3_	[40]
			775	Indicates the existence of –CN and –C–Cl stretching vibrations	[37]
-	750	-	-	CH_2_ bending	[41]

#### 3.1.2. Crosslinking Effect in Hydrogels

**Figure 2:**
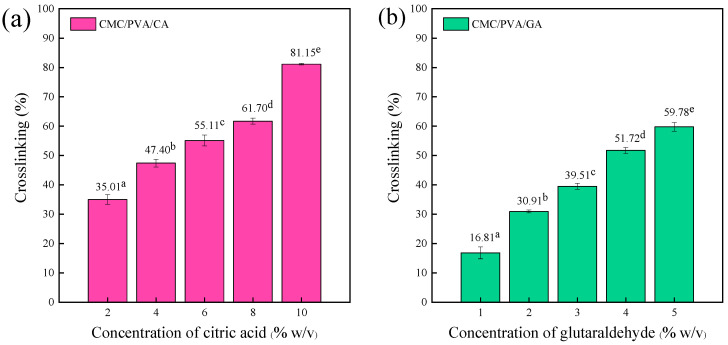
Crosslinking percentage from the measurement of (**a**) CMC/PVA/CA and (**b**) CMC/PVA/GA hydrogel film conditions.

The CMC/PVA hydrogel was treated with varying concentrations of CA additive agents (2–10% *w*/*v*), and the crosslinking percentage was examined, as shown in Figure 2a. Similarly, different GA concentrations (1–5% *w*/*v*) were also used to improve the crosslinking percentage in CMC/PVA. It was found that the crosslinking agent properties of hydrogels corresponded with the pore size in hydrogel films, depending on the type and concentration of crosslinking agents (CA and GA). As a result, Figure 2a reveals the increment of crosslinking percentage in CMC/PVA/CA for 35.01 to 81.51% (a–e), which is influenced by adding CA concentrations corresponding to 2–10% *w*/*v*. CMC/PVA/GA also indicated the crosslinking percentage increase from 16.81 to 59.78% (a–e), which is implicated by the overfilled higher GA concentrations for 1–5% *w*/*v*, as shown in Figure 2b. However, the crosslinking percentage of both hydrogels implied an increased development of porous capability on hydrogel films, correlating with the swelling properties of hydrogels [44]. Thus, the morphology of both hydrogels with and without crosslinking from CA and GA was investigated.

In addition, the degree of crosslinking in a polymer network structure is mainly influenced by the molecular weight between the crosslinks, which significantly dictates the different properties of a polymer gel, such as mechanical strength and swelling ratio. Figure 2a shows the crosslinking percentage from CMC/PVA/CA hydrogel films with CA added, obtained by ester bonding. As a result, the highest crosslinking percentage with CA was indicated at 10% *w*/*v* of CA, where the crosslinking percentage reached 81% and decreased with a lower CA concentration. The CA crosslinking agent showed an ester crosslinking [19] containing the –OH functional group, corresponding to the solubility properties of hydrogel films, as explained in the next section. Figure 2b shows the increment of the crosslinking percentage with adding higher GA concentrations, indicating that a higher concentration of the GA additive agent induced the crosslinking percentage depending on the GA concentrations based on the ether bond. A high crosslinking percentage from adding GA with 5% *w*/*v* showed that the maximum crosslink was 60%.

#### 3.1.3. Morphology of Hydrogels

The effect of crosslinking properties on hydrogel properties was affected by the porous ability of hydrogels, as indicated by the comparison of the morphology of CMC/PVA/CA and CMC/PVA/GA hydrogels. Thus, the SEM results demonstrated the significance of the properties of hydrogels with and without crosslinking from CA and GA through the cross-sections of the hydrogel films, presenting pore structures [26]. Similarly, the CMC/PVA hydrogel’s morphology was not very porous, with a slightly rough surface, before being treated with additive crosslink agents.

**Figure 3:**
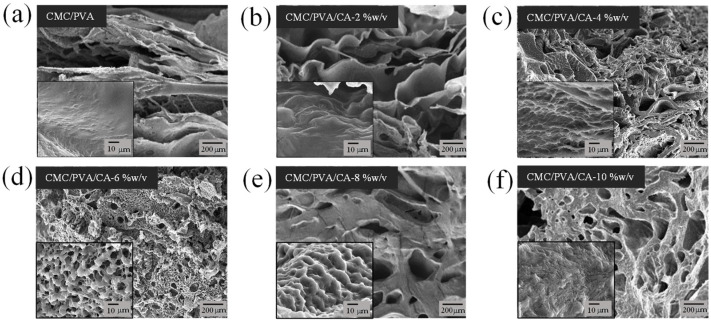
SEM morphology of the CMC/PVA hydrogel films and various CA concentrations. (**a**) CMC/PVA, (**b**) CMC/PVA/CA-2% *w*/*v*, (**c**) CMC/PVA/CA-4% *w*/*v*, (**d**) CMC/PVA/CA-6% *w*/*v*, (**e**) CMC/PVA/CA-8% *w*/*v*, and (**f**) CMC/PVA/CA-10% *w*/*v*.

**Figure 4:**
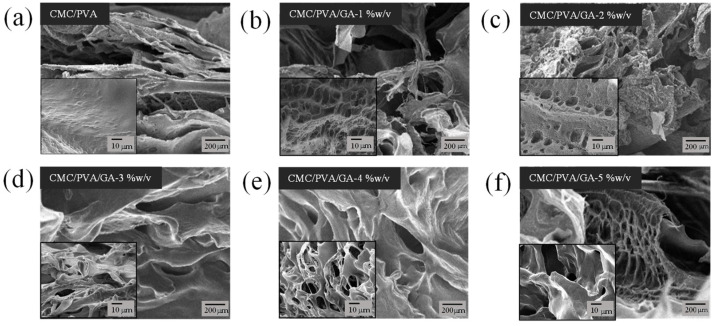
SEM morphology of the CMC/PVA hydrogel films and various GA concentrations, (**a**) CMC/PVA, (**b**) CMC/PVA/GA-1% *w*/*v*, (**c**) CMC/PVA/GA-2% *w*/*v*, (**d**) CMC/PVA/GA-3% *w*/*v*, (**e**) CMC/PVA/GA-4% *w*/*v*, and (**f**) CMC/PVA/GA-5% *w*/*v*.

Afterwards, CMC/PVA was treated with various CA and GA concentrations, displaying the development of surface roughness on hydrogel films caused by the porous ability of each hydrogel condition. As the SEM results show, CMC/PVA/CA revealed a highly porous structure after crosslinking CMC/PVA with a CA additive agent at concentrations of 2–10% *w*/*v*, as shown in Figure 3b–f. Interestingly, the increase in surface roughness for CMC/PVA/CA depended on the addition of CA from a concentration of 2% *w*/*v* up to a maximum concentration of 6% *w*/*v*. Thus, CMC/PVA/CA-6% (Figure 3d) exhibited a highly porous morphology and a highly dense crosslink structure on the hydrogel. The highly porous structure of the hydrogel supported the diffusion of water molecules when the hydrogel films were placed in an aqueous medium. It was found that when the concentration of the crosslinking substance reached a certain point, the pores of the hydrogels were narrowed by the coagulation of crosslinking agents in different CA concentrations. Hence, the crosslinking concentrations affected the pore characteristics in the CMC/PVA/CA hydrogel films (Figure 3). Meanwhile, CMC/PVA/GA in Figure 4a–f exhibits crosslinking with different GA concentrations (1–5%), influencing the porousness of CMC/PVA/GA hydrogel films. It indicated that CMC/PVA with the addition of 1–2% *w*/*v* of GA induced porosity in CMC/PVA/GA by forming a crosslinking structure with the GA agent. However, all CMC/PVA/GA conditions were still different from CMC/PVA, which is easy to observe compared to CMC/PVA/CA. Therefore, it was also observed that the type and concentration of crosslinking agents influenced the pore size of hydrogel films. Based on the SEM results, the overall pore size (Figures S1 and S2) of the CMC/PVA hydrogel with the CA crosslinking agent was around 18.41 to 69.98 m, which was larger than that of the CMC/PVA hydrogel with the GA crosslinking agent, which was around 3.57 to 17.17 μm. Therefore, a larger pore size in hydrogels implies greater swelling and a higher water absorption capacity.

### 3.2. Modification of Hydrogel Properties

#### 3.2.1. Swelling Properties of Hydrogels

**Figure 5:**
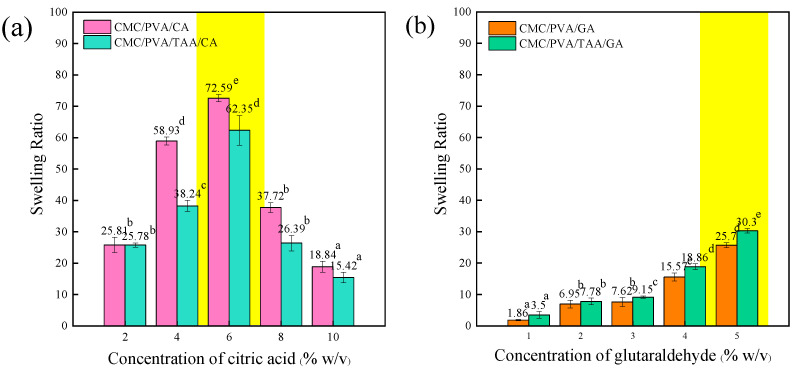
Comparison of the swelling properties from crosslink agent between (**a**) CA and (**b**) GA in CMC/PVA hydrogel film conditions combined with TAA in a component (yellow highlight: the highest swelling ratio).

In Figure 5, the swelling properties of hydrogels were inspected in CMC/PVA, CMC/PVA/CA, and CMC/PVA/GA, comparing the influence of crosslinking with swelling properties in each condition. As a result, the swelling ratio CMC/PVA/CA was enhanced from 2 to 6% *w*/*v*, exhibiting the maximum swelling ratio at 72.53% and reducing with higher concentrations of the CA additive agent from 8 to 10% *w*/*v* (18.84–37.72% of the swelling ratio). A higher CA concentration (8–10% *w*/*v*) reduced swelling ratio properties due to the excess crosslinking between hydrogel films and CA additive agents [45]. Meanwhile, adding TAA into CMC/PVA/CA demonstrated the reduction of swelling ratio properties for all conditions of the CA additive [46], as depicted in Figure 5a. With the addition of GA, the swelling properties of the CMC/PVA/GA film were increased, depending on the weight of the GA additive (1–5% *w*/*v*), as shown in Figure 5b. Meanwhile, the addition of higher GA concentrations increased the swelling ratio of hydrogel films from 1.86 to 25.7% (a–e). In contrast, CMC/PVA/GA was treated with TAA, demonstrating an improved swelling ratio in CMC/PVA/GA/TAA hydrogels from 3.5 to 30.3% with higher CA concentrations. The higher swelling ratio of the GA additive corresponded with the crosslinking percentage in the hydrogel films, but it was still low compared to the swelling ratio with the CA additive. This implies that the CA additive in the CMC/PVA was compatible with the hydrogel structure and showed the highest swelling ratio properties compared to the GA additive. Based on the swelling results between hydrogels with CA and GA, the swelling of hydrogels with CA exhibited significant crosslinked materials arising primarily from the interaction between CA and the residual free C=O groups inherent in the polymer structure. During hydrogel formation, the ester bonds were generated by the reactions between C=O groups from CA and -OH polymer groups. Meanwhile, CA is composed of a trifunctional nature of three C=O groups and acts as an effective crosslinking agent by undergoing esterification reactions with -OH groups in the polymer chains [9], leading to more residual C=O groups. Thus, the high C=O group content in CA post-crosslinking induced a high water propensity in the hydrogel, resulting in more swelling than GA. Similarly, the presence of unreacted C=O groups post-crosslinking interacted with water molecules, influencing the high capacity of the hydrogel to absorb water and significantly affecting its swelling behavior [8]. Thus, there is a significant difference between the swelling extent of hydrogels crosslinked with CA and those crosslinked with GA, which was attributed to the presence and concentration of residual free C=O groups resulting from the CA crosslinking process.

#### 3.2.2. Hydrogel Solubility Properties

**Figure 6:**
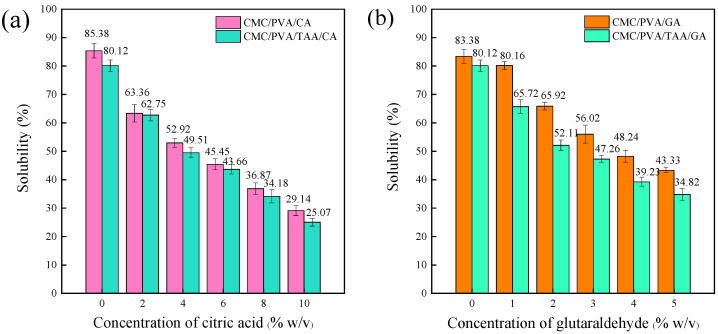
Solubility percentage of (**a**) CMC/PVA/CA and CMC/PVA/CA/TAA and (**b**) CMC/PVA/GA and CMC/PVA/GA/TAA hydrogel films.

The enhanced solubility of poorly water-soluble drugs is an essential factor in preparing effective dosage forms that can be administered systemically or applied locally. The size and morphology of the drug affect its biopharmaceutical properties, such as solubility [47]. It is indicated that the higher CA additive concentrations (2 to 10%) decreased the solubility of CMC/PVA/CA from 63.36 to 23.14% (Figure 6a), which was influenced by the increase in crosslinking percentage in the hydrogel. Similarly, the solubility of CMC/PVA/GA in Figure 6b decreased from 80.16 to 43.33% with higher concentrations of GA (1–5% *w*/*v*). Thus, CMC/PVA/GA indicated a higher crosslinking percentage, but the solubility of hydrogels was reduced with the inverse relationship. Figure 6a,b significantly compares the solubility of CMC/PVA/CA and CMC/PVA/GA hydrogel films, revealing an inverse relationship with the increment of crosslinking concentration. Consequently, the enhanced soluble content of poorly water-soluble drugs is an essential factor in preparing effective dosage forms that can be administered systemically or applied locally [6]. When TAA was added, CMC/PVA/CA/TAA and CMC/PVA/GA/TAA hydrogel films exhibited an overall solubility similar to that of hydrogels treated with the additive agents before adding TAA, which revealed a slightly reduced solubility, as shown in Figure 6a,b.

In this study, a solubility test is associated with in vitro biodegradability, promoting complementary methodologies for evaluating the properties of materials intended for biomedical use. This integrated approach enhances the understanding of material characteristics and facilitates improving and customizing materials for specific biomedical applications. In previous research, a solubility test in the CMC/PVA hydrogel was performed to examine the solubility of norfloxacin [48], demonstrating the importance of solubility testing for influencing in vitro biodegradability. Similarly, our work determined that CMC/PVA hydrogel films containing CA and GA contribute to their biodegradability, as indicated by the decrease in solubility with the increased concentration of both crosslinking agents, as illustrated in Figure 6. Thus, the reduction in solubility enabled the hydrogel to retain and release the drug gradually, supporting the drug-releasing feature application of the hydrogel.

#### 3.2.3. Water Retention Capacity of Hydrogels

**Figure 7:**
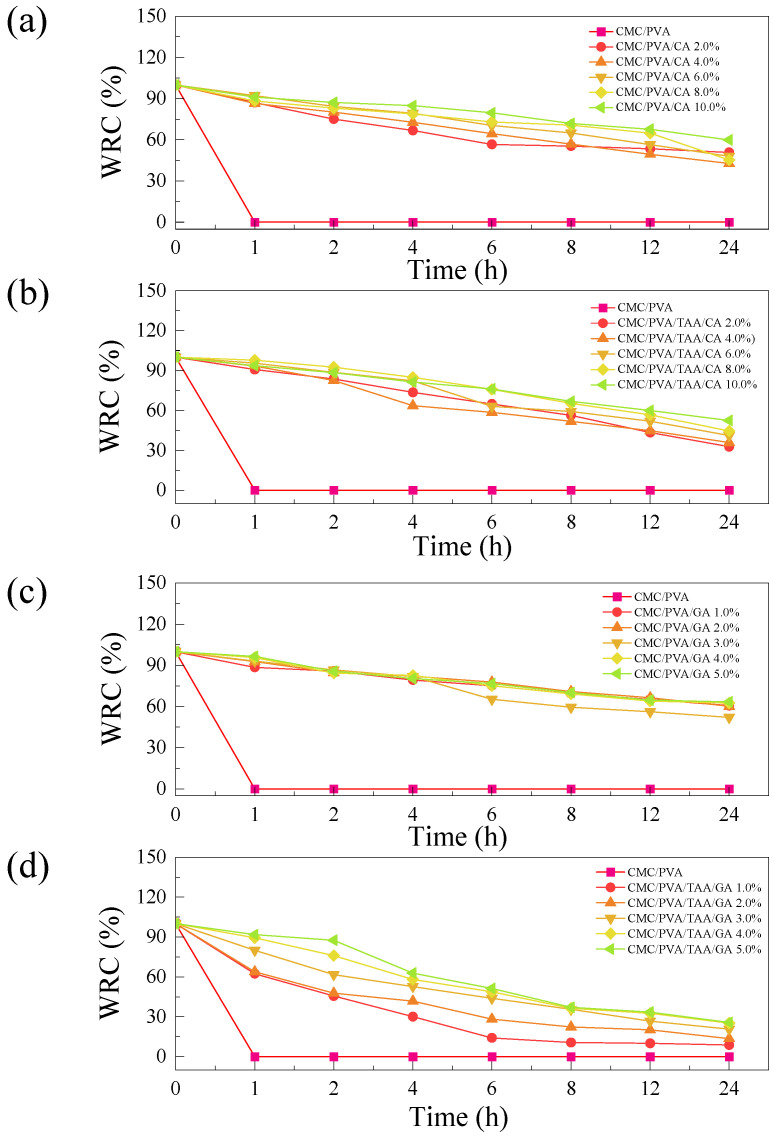
WRC of CMC/PVA hydrogel films incorporated with (**a**) CA, (**b**) TAA/CA, (**c**) GA, and (**d**) GA/TAA.

The capability of CMC/PVA hydrogel films to hold water was observed in the hydrogel films, defined via water retention capacity (WRC) within 24 h. Figure 7a displayed the WRC of CMC/PVA hydrogel films, showing that water was retained for only 1 h and subsequently lost. For conditions where CA was added, CMC/PVA/CA revealed the effectiveness of WRC in CMC/PVA/CA within 24 h, with the highest and lowest WRC results at CMC/PVA/CA-10% *w*/*v* and CMC/PVA/CA-2% *w*/*v*, respectively. With the addition of 2% *w*/*v* CA in CMC/PVA/CA, it was revealed that the WRC reduced after 24 h and remained at 50%. It remained at 43% when 4% *w*/*v* CA was added into CMC/PVA. In addition, a higher CA weight at 6 and 8% *w*/*v* of the CA additive indicated a WRC of 48 and 45%, respectively. Interestingly, the highest WRC of 60% was reported at 10% *w*/*v* of the CA additive.

With the same WRC testing approach, Figure 7b shows the corresponding WRC based on the addition of TAA into CMC/PVA/CA, indicating compatible hydrogel features for drug testing. The WRC trends were exposed to be similar to CMC/PVA/CA at various CA weight concentrations. Thus, CMC/PVA/CA/TAA hydrogel films exhibited the highest WRC, remaining at 52% after 24 h after adding CA at a concentration of 10% *w*/*v* before reducing to 44, 41, 36, and 35% after adding 8, 6, 4, and 2% *w*/*v* of the CA additive, respectively.

Furthermore, the effect of different GA concentrations (1–5% *w*/*v*) on the WRC of the CMC/PVA showed that the highest WRC results within 24 h at 63% were observed for the CMC/PVA incorporated with 5% *w*/*v* of GA (Figure 7c), decreasing to 62, 52, 51, and 50% when adding 4, 3, 2, and 1% *w*/*v* of GA, respectively. Interestingly, Figure 7d shows the WRC feature of CMC/PVA/GA applied with TAA, exhibiting the significance of TAA in CMC/PVA/GA with various GA concentrations. It was concluded that the increase in WRC depended on a higher GA concentration, corresponding to the WRC results in Figure 7d. A higher concentration of GA at 5% *w*/*v* enhanced the WRC, which reached 26% within 24 h of testing. In contrast, the decreased GA impacted the leveling of the WRC by reducing concentrations from 25% (4% *w*/*v* of GA) to 8% (1% *w*/*v* of GA), as shown in Figure 7d.

The WRC results demonstrated the influence of crosslinking agents [21], improving the WRC of the hydrogel films [42]. Adding CA and GA additive agents to CMC/PVA demonstrated outstanding WRC results under CMC/PVA/CA-10% *w*/*v* and CMC/PVA/GA-6% *w*/*v* conditions compared to CMC/PVA, showing a maximum WRC of only 1 h. Thus, the addition of CA and GA crosslinking agents significantly impacted the water-holding properties, with a higher WRC developed for usage in drug-releasing properties on CMC/PVA hydrogels.

#### 3.2.4. Release Ability of Anti-Inflammatory Drugs (In Vitro)

The release ability of anti-inflammatory drugs (in vitro) based on the modified CMC/PVA hydrogel films with CA and GA additive agents was investigated through the release ability of TAA within 24 h (% Released TAA), using the UV–vis technique.

**Figure 8:**
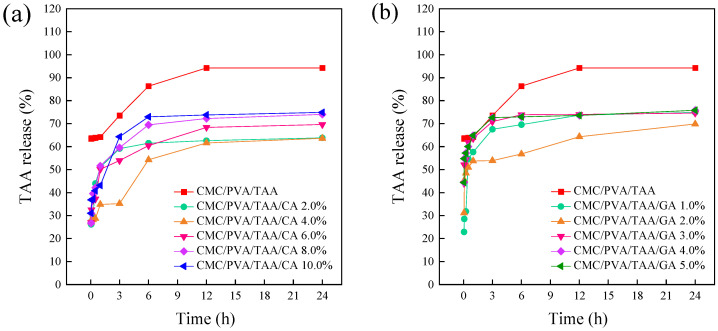
Drug release ability of CMC/PVA/TAA with different concentrations of (**a**) CA and (**b**) GA additive crosslinked agents.

The optimized releasing conditions were tested with PBS (pH 7.4) at 37 °C [12] and treated onto the modified CMC/PVA hydrogel conditions, as shown in Figure 8. For the CMC/PVA conditions, the release ability of TAA is presented in Figure 8a, which displays the TAA-releasing phenomenon from the modified CMC/PVA with various CA concentrations (2–10% *w*/*v*). As the crosslinking of the CA additive was added to CMC/PVA, it improved the drug release from hydrogel films by increasing the CA additive concentration, correlating to the swelling properties of CMC/PVA from adding higher CA concentrations, as explained in the previous section. According to the TAA-releasing results, CMC/PVA/CA exposed outstanding effectiveness in significantly holding and releasing TAA compared to CMC/PVA properties at the same time of duration in testing. As a result, it was indicated that CMC/PVA in Figure 8a was rapidly decomposed during the TAA-releasing test, implying CMC/PVA cannot hold the drug and is immediately released. Meanwhile, the TAA-releasing results of CMC/PVA/CA in 24 h demonstrated the highest percentage of the TAA release rate at 75% in CMC/PVA/CA-10% *w*/*v*/TAA, then decreased to 70 and 50% for CA additives at 6 and 4% *w*/*v*, respectively. Interestingly, CMC/PVA/CA showed an improvement in drug-release properties in hydrogel films under higher CA concentrations and slowly controlled the TAA release rate within 24 h, which was affected by the increased crosslinking in the structure.

Moreover, GA (1–5% *w*/*v*) was added to CMC/PVA, revealing the maximum increment of the crosslinker in CMC/PVA/GA with a concentration of 4 to 5% *w*/*v*, improving the efficacy of the drug release rate up to 75%, as shown in Figure 8b. In contrast, adding GA of less than 3% demonstrated a TAA release of 50%, similar to CMC/PVA in control conditions and not significantly different to the overall TAA release.

Figure 8a,b shows that the overall percentage of the release rate of hydrogel films increased over time under controlled conditions. Consequently, the TAA-releasing results of this experiment corresponded with adding the crosslinking agent, which improved the drug release rate more effectively than non-crosslinking in hydrogel films.

**Figure 9:**
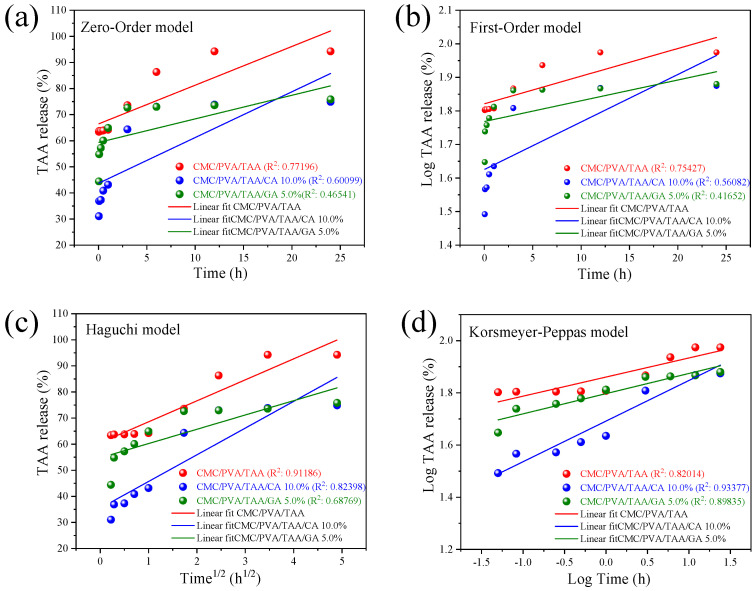
TAA release data fitted with different kinetic models, the (**a**) zero–order, (**b**) first–order, (**c**) Higuchi, and (**d**) Korsmeyer–Peppas models.

For the kinetics analysis, the TAA release was tested on the modified CMC/PVA hydrogel with CA and GA crosslink agents and on the hydrogel without crosslink agents with various kinetic models, such as the zero-order, first-order, Higuchi, and Korsmeyer–Peppas models [28,29], as presented in Figure 9. It was indicated that the Korsmeyer–Peppas drug diffusion model (Figure 9d) displayed a linear relationship fitting of the TAA-releasing process, corresponding to the TAA-releasing times of the hydrogel in this work. Based on the data-fitted kinetics model (Table 3), CMC/PVA/CA-10%/TAA exhibited data fitted to a linear relationship with the highest R^2^ at 0.93377, which is higher than the hydrogels under CMC/PVA/GA-5%/TAA and CMC/PVA/TAA conditions, which had R^2^ values of 0.89835 and 0.82014, respectively. Thus, the Korsmeyer–Peppas kinetic model was considered to correspond to the kinetic models of TAA released for the hydrogel in this work.

For future investigations, the hydrogel will be subject to in vivo studies to ascertain its biocompatibility, effectiveness in drug delivery, and prospective therapeutic uses. The delivery of anti-inflammatory drugs and potentially other therapeutic agents is envisioned for our hydrogel. Notably, the attributes of the hydrogel include the capacity to encapsulate and release drugs in a controlled manner, rendering it suitable for targeted drug delivery applications, improving precise drug release and being advantageous for wound healing or regenerative medicine contexts. Furthermore, the hydrogels exhibit favorable physicochemical properties, cytocompatibility, and hemocompatibility, positioning them as promising candidates for smart wound dressings in skin tissue engineering. This innovative approach represents a significant advancement toward addressing the challenges associated with chronic wound management and applications in skin tissue engineering [49].

## 4. Conclusions

In summary, this work successfully prepared crosslinked CMC/PVA hydrogel films with enhanced hydrogel patching ability for wound healing in medical treatment using different concentrations of GA and CA additive agents. Interestingly, the addition of crosslinkers improved swelling and drug-releasing capability compared to non-crosslinked hydrogel films. Thus, the swelling property of hydrogels revealed an outstanding property of hydrogels, with drug-releasing capability within 24 h. Notably, the modified hydrogel films demonstrated TAA absorption, corresponding to the increased crosslinker density, which can be used to delay drug-release time, improving the efficiency of TAA in anti-inflammatory treatment. Consequently, hydrogel films in this work showed the capability of being an alternative material in enhanced anti-inflammatory wound healing and can be used to develop medical materials from natural materials that are biocompatible and biodegradable.

## Figures and Tables

**Table 2:** FTIR peak assignments and their corresponding functional groups observed in CMC/PVA, CMC/PVA/CA, and CMC/PVA/GA.

Wavenumber (cm^−1^)			Characteristic Bond and Movement	Ref.
CMC/PVA	CMC/PVA/CA	CMC/PVA/GA		
-	3700–3400	-	O–H stretching vibration (Chain end)	[42]
3340			O–H stretching vibration (Chain end)	[24]
-	3000–2800	3000–2800	O–H stretching vibration in CH, CH_2_, CH_3_	
2922	-	-	C–H stretching vibration	[36]
-	1740	1740	C=O stretching vibration	[9]
1654	-	-	C=O stretching vibration	[36]
-	-	1627	Corresponds to –C=O, should be a sign of the excess of GA	[37]
1422	1425	1425	C–H bending in CH_2_, CH_3_	[31]
1325	1325	1325	C–H bending in CH, CH_2_, CH_3_	[31]
-	1081	1083	Corresponds to C-O-C stretching	[43]
-	-	1060	C–O–C bending	[37]
-	-	1058	C–O stretching vibration	[43]
839	839	839	C–H bending in CH, CH_2_, CH_3_	[31]
750	750	750	CH_2_ bending	[36]

**Table 3:** Kinetic model parameters for TAA release from the modified CMC/PVA hydrogels.

Hydrogels	pH	Zero–Order (R^2^)	First–Order (R^2^)	Higuchi (R^2^)	Korsmeyer–Peppas (R^2^)
CMC/PVA/TAA	7.4	0.77196	0.75427	0.91186	0.82014
CMC/PVA/CA-10%/TAA	7.4	0.60099	0.56082	0.82398	0.93377
CMC/PVA/GA-5%/TAA	7.4	0.46541	0.41652	0.68769	0.89835

## Data Availability

The original contributions presented in the study are included in the article and Supplementary Materials, further inquiries can be directed to the corresponding authors.

## Supplementary Materials

The following supporting information can be downloaded at: https://www.mdpi.com/article/10.3390/polym16131798/s1, Figure S1a–d: we already update Pore size distribution of the modified CMC/PVA hydrogel with CA crosslinking agent; Figure S2a–e: Pore size distribution of the modified CMC/PVA hydrogel with GA crosslinking agent; Table S1: The compositions of CMC/PVA hydrogel films containing different concentrations of CA and GA; Table S2: The compositions of CMC/PVA hydrogel films containing different concentrations of CA, GA, and TAA.
