# Supplementary material for: Modification of a Carboxymethyl Cellulose/Poly(vinyl alcohol) Hydrogel Film with Citric Acid and Glutaraldehyde Crosslink Agents to Enhance the Anti-Inflammatory Effectiveness of Triamcinolone Acetonide in Wound Healing
Source: Polymers (Basel). 2024 Jun 25;16(13):1798. doi: 10.3390/polym16131798 (PMC11244469; doi:10.3390/polym16131798)
Supplement: Supplementary file 1 [file polymers-16-01798-s001.zip › polymers-3033985-supplementary.pdf]

polymers

MDPI

Supplementary Materials

Modification of a Carboxymethyl Cellulose/Poly(vinyl alcohol) Hydrogel Film with Citric Acid and Glutaraldehyde Crosslink Agents to Enhance the Anti-Inflammatory Effectiveness of Triamcinolone Acetonide in Wound Healing

Kanticha Pratinthong [1], Winita Punyodom [2,3,*], Pensak Jantrawut [4,5], Kittisak Jantanasakulwong [1,3,5], Wirongrong Tongdeesoontorn [6,7], Montira Sriyai [8,9], Rangsan Panyathip [1,8], Sarinthip Thanakkasaranee [1,3,5], Patnarin Worajittiphon [2,3], Nuttapol Tanadchangsaeng [10] and Pornchai Rachtanapun [1,3,5,*]

1 Division of Packaging Technology, School of Agro-Industry, Faculty of Agro-Industry, Chiang Mai University, Chiang Mai 50100, Thailand; kanticha_p@cmu.ac.th (K.P.); kittisak.jan@cmu.ac.th (K.J.); rangsanpanyatip@gmail.com (R.P.); sarinthip.t@cmu.ac.th (S.T.)
2 Department of Chemistry, Faculty of Science, Chiang Mai University, Chiang Mai 50200, Thailand; patnarin.w@cmu.ac.th
3 Center of Excellence in Materials Science and Technology, Faculty of Science, Chiang Mai University, Chiang Mai 50200, Thailand
4 Department of Pharmaceutical Sciences, Faculty of Pharmacy, Chiang Mai University, Muang, Chiang Mai 50200, Thailand; pensak.j@cmu.ac.th
5 Center of Excellence in Agro Bio-Circular-Green Industry (Agro BCG), Chiang Mai University, Chiang Mai 50100, Thailand
6 School of Agro-Industry, Mae Fah Luang University, 333 Moo 1 Tasud, Chiang Rai 57100, Thailand; wirongrong.ton@mfu.ac.th
7 Research Center of Innovative Food Packaging and Biomaterials Unit, Mae Fah Luang University, 333 Moo 1 Tasud, Chiang Rai 57100, Thailand
8 Office of Research Administration, Chiang Mai University, Chiang Mai 50200, Thailand; montirasriyai@gmail.com
9 Bioplastics Production Laboratory for Medical Applications, Faculty of Science, Chiang Mai University, Chiang Mai 50200, Thailand
10 College of Biomedical Engineering, Rangsit University, Pathumthani 12000, Thailand; nuttapol.t@rsu.ac.th
* Correspondence: winitacmu@gmail.com (W.P.); pornchai.r@cmu.ac.th (P.R.); Tel.: +66-814-810-781 (W.P); +66-635-492-556 (P.R.)

Table S1. The compositions of CMC/PVA hydrogel films containing different concentrations of CA and GA.

Bath	CMC (%w/v)	PVA (%w/v)	CA (%)	GA (%)
**1**			-	-
**2**			2.0	-
**3**			4.0	-
**4**			6.0	-
**5**			8.0	-
**6**	2.0	2.0	10.0	-
**7**			-	1.0
**8**			-	2.0
**9**			-	3.0
**10**			-	4.0
**11**			-	5.0

Table S2. The compositions of CMC/PVA hydrogel films containing different concentrations of CA, GA, and TAA.

Bath	CMC (%w/v)	PVA (%w/v)	CA (%)	GA (%)	TAA (%)
**12**			-	-	
**13**			2.0	-	
**14**			4.0	-	
**15**			6.0	-	
**16**			8.0	-	
**17**	2.0	2.0	10.0	-	0.1
**18**			-	1.0	
**19**			-	2.0	
**20**			-	3.0	
**21**			-	4.0	
**22**			-	5.0	

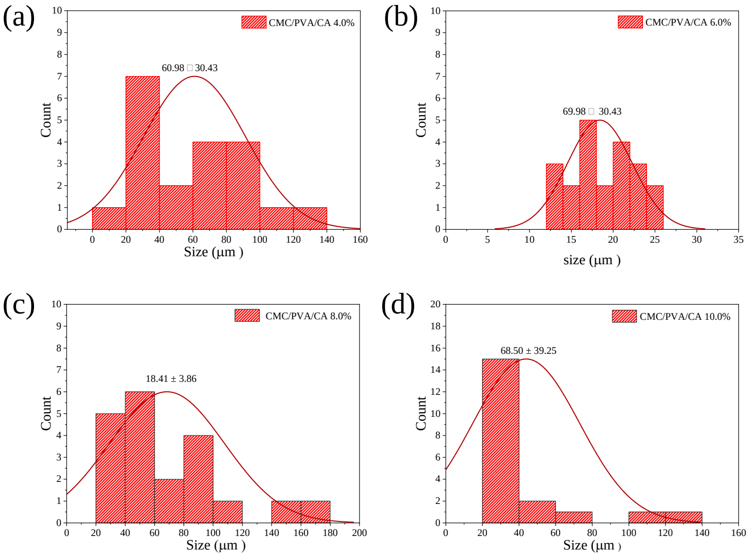

Figure S1. Pore size distribution of the modified CMC/PVA hydrogel with CA crosslinking agent.

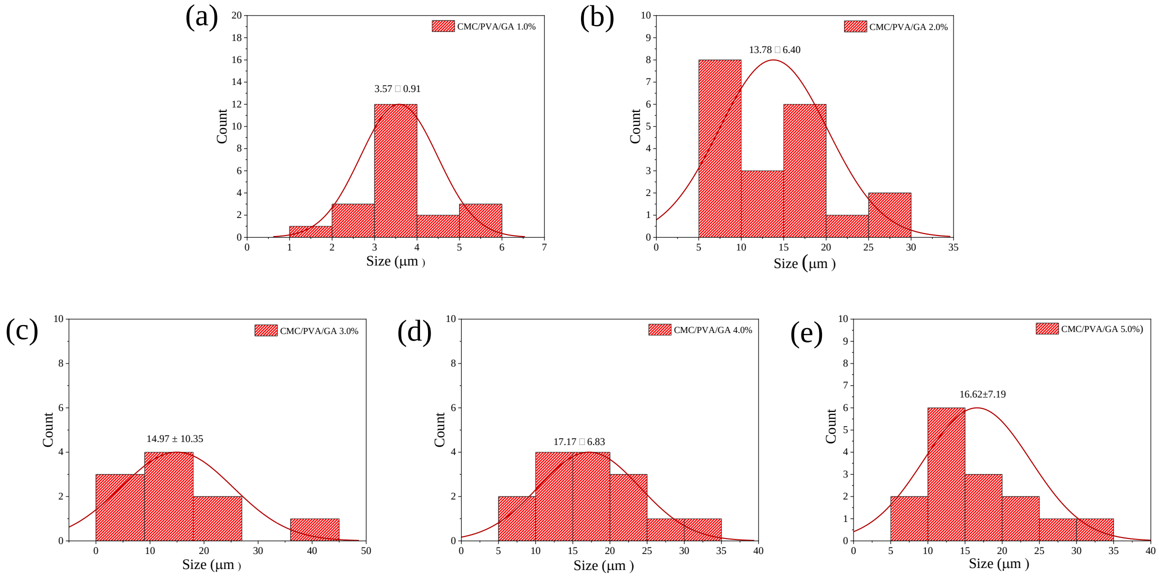

Figure S2. Pore size distribution of the modified CMC/PVA hydrogel with GA crosslinking agent.

Disclaimer/Publisher's Note: The statements, opinions and data contained in all publications are solely those of the individual author(s) and contributor(s) and not of MDPI and/or the editor(s). MDPI and/or the editor(s) disclaim responsibility for any injury to people or property resulting from any ideas, methods, instructions or products referred to in the content.
